# Synthesis and Characterization of In-Situ-Prepared Nanocomposites Based on Poly(Propylene 2,5-Furan Dicarboxylate) and Aluminosilicate Clays

**DOI:** 10.3390/polym10090937

**Published:** 2018-08-23

**Authors:** Lazaros Papadopoulos, Zoi Terzopoulou, Dimitrios N. Bikiaris, Dimitra Patsiaoura, Kostantinos Chrissafis, Dimitrios G. Papageorgiou, George Z. Papageorgiou

**Affiliations:** 1 Laboratory of Polymer Chemistry and Technology, Department of Chemistry, Aristotle University of Thessaloniki, GR-541 24 Thessaloniki, Greece; lazaros.geo.papadopoulos@gmail.com (L.P.); terzozoi@chem.auth.gr (Z.T.); dbic@chem.auth.gr (D.N.B.); 2 Solid State Physics Section, Physics Department, Aristotle University of Thessaloniki, GR-541 24 Thessaloniki, Greece; dpatsi@physics.auth.gr (D.P.); hrisafis@physics.auth.gr (K.C.); 3 School of Materials and National Graphene Institute, University of Manchester, Oxford Road, Manchester M13 9PL, UK; dimitrios.papageorgiou@manchester.ac.uk; 4 Department of Chemistry, University of Ioannina, P.O. Box 1186, GR-45110 Ioannina, Greece

**Keywords:** biobased polyesters, poly(propylene 2,5-furan dicarboxylate), nanocomposites, aluminosilicate clays

## Abstract

Poly(propylene 2,5-furan dicarboxylate) (PPF), or poly(trimethylene 2,5-furan dicarboxylate) (PTF), is a biobased alipharomatic polyester that is expected to replace its fossil-based terephthalate (PPT) and naphthate (PPN) homologues. PPF possesses exceptional gas barrier properties, but its slow crystallization rate might affect its success in specific applications in the future. Therefore, a series of PPF based nanocomposites with the nanoclays Cloisite^®^-Na (MMT), Cloisite^®^-20A (MMT 20A), and halloysite nanotubes (HNT) were synthesized via the in situ transterification and polycondensation method. The effect of the nanoclays on the structure, thermal, and crystallization properties of PPF was studied with several methods including infrared spectroscopy (IR), Nuclear Resonance Spectroscopy (^1^H-NMR), Wide Angle X-ray Diffraction (WAXD), Thermogravimetric Analysis (TGA), and Differential Scanning Calorimetry (DSC). The insertion of the nanofillers in the polymer matrix altered the crystallization rates, and TGA results showed good thermal stability, since no significant mass loss occurred up to 300 °C. Finally, the degradation mechanism was studied in depth with Pyrolysis-Gas Chromatography/Mass Spectroscopy, and it was found that β-scission is the dominant degradation mechanism.

## 1. Introduction

In recent years, the possible replacement of fossil fuels for producing monomers from cheap and renewable raw materials such as cellulose, starch, lignin, proteins, and vegetable oils is being extensively explored in order to develop a more sustainable “green” economy. In this context, bio-based polymers (polymers derived from renewable sources) have attracted the interest of industries and consumers around the world over the last 20 years. At the same time, European and American legislative frameworks change in favor of biobased products and at the expense of petrochemicals [1,2]. The bio-based products sector is characterized as a key enabling technology by the E.U. [3], and a priority area for development and funding.

One of the biomass-derived monomers that stands out in both academic and industrial research is 2,5-furandicarboxylic acid (FDCA). FDCA is produced by biomass from a series of sugar dehydration reactions towards hydroxymethylfurfural and its subsequent oxidation, and is mainly used for the production of biobased polyesters. Fully biobased polyesters can be synthesized from FDCA when combined with biobased diols that are already being produced on an industrial scale. The FDCA-based polyester with the greatest interest is poly(ethylene 2,5-furan dicarboxylate) (PEF), since it is believed it will replace poly(ethylene terephthalate) (PET) in packaging applications as its biobased homologue. In that direction, the effect of several catalysts [4], as well as of solid state polymerization on the molecular weight increase of PEF, was recently studied [5,6,7].

Furanic polyesters with various other diols besides ethylene glycol have been synthesized and studied recently [8,9], one of them being poly(propylene 2,5-furan dicarboxylate) (PPF), derived from the transesterification and polycondensation of FDCA with 1,3-propanediol (PDO) [10,11,12]. The spark on the rise of production and the use of PDO-based polymers came with the beginning of PDO production from renewable sources [13], with DuPont and Tate & Lyle opening the world’s first PDO production plant from corn sugar [14]. DuPont uses biobased PDO for the synthesis of soft and resilient poly(propylene terephthalate) fibers under the trademark name Sorona^®^ that are being used in the textile and carpet industry [15]. A number of patents concerning PPF were published by the same company [16,17,18,19] that highlight the excellent gas barrier properties of the polyester, making it an attractive material for multilayered packaging materials with a very high permeation barrier. DuPont has achieved the production of PPF with acceptable color and is aiming on marketing it towards the packaging industry [20,21,22]. Compared to its fossil-based counterpart poly(propylene terephthalate) (PPT), PPF shows a higher glass transition temperature (*T*_g_ = 57 °C for PPF compared to 47 °C for PPT); lower melting temperature (*T*_m_ = 180 °C for PPF vs. 226 °C for PPT); slower crystallization rates, as a result of the narrower temperature window for crystallization; a lower crystallization temperature (*T*_c_) with very small spherulites; and a slightly lower thermal stability [11]. While its lower *T*_c_ and *T*_c_ are considered advantages concerning energy consumption during melt processing, its slow crystallization rate can affect the achievement of specific properties for some applications.

Polymer-based nanocomposites are a class of materials that constitute a polymeric matrix along with nanosized fillers, with superior properties such as mechanical, thermal, electrical, and gas barriers, depending on the application. Nanoparticles can also strongly affect the crystallization of polymeric matrices, as they can substitute the absence of primary nuclei [23]. Clays are a very common type of natural reinforcing nanofillers, composed of silica, alumina, and water, with a particle size ranging from 10–100 nm [24]. Montmorillonite (MMT) is a hydrophilic, layered nanoclay that consists of “platelets with an inner octahedral layer sandwiched between two silicate tetrahedral layers” [23]. It has been widely studied and used as a filler in paints and rubbers; as a plasticizer; and in the preparation of electrical-, heat-, and acid-resistant porcelain during the last 20 years [25]. Their hydrophilic nature makes MMTs incompatible with hydrophobic polymers, so usually the Na^+^ cation present between its layers is exchanged with organic cations, such as alkylammonium or alkyphosphonium/onium ions [26]. Halloysite nanotubes (HNT) are another type of natural multiwalled nanoclays that comprise aluminosilicate sheets rolled into tubes, with the molecular structure Al_2_Si_2_O_5_(OH)_4_·nH_2_O. Because of its unique morphology, with most aluminol groups present on the inner surface and siloxane groups on the outer surface, HNTs are relatively hydrophobic, making their dispersion in non-polar polymers easier [27].

Polymer nanocomposites can be synthesized mainly via melt mixing, solution casting, or in situ polymerization. In contrast with the first two methods, in situ polymerization allows the formation of strong, covalent interactions between the nanofillers and the polymeric matrix, because it involves the dispersion of the clay into the monomer, which is afterwards polymerized, providing it with an additional role: that of a catalyst [28]. Moreover, it involves the swelling of the clay layers in the liquid monomer and can result in either intercalated or partially exfoliated nanocomposite structures [29].

So far, only two studies on nanocomposites of furan-based polyesters with nanoclays have been published. Martino et al. prepared PEF nanocomposites with 2 wt % and 4 wt % montmorillonite modified with either dimethyl benzyl hydrogenated tallow alkyl or octadecylamine and organophilic sepiolite by melt extrusion and compression molding [30]. The presence of these clays in PEF decelerated its thermal degradation and increased its glass transition temperature (*T*_g_) slightly, while their morphology was mainly exfoliated. The same group also prepared the same nanocomposites with the solvent casting method [31], in which the nanoclays were predominantly intercalated, which did not influence the *T*_g_ and slightly accelerated the crystallization rate of PEF. From both studies, it was concluded that melt processing allowed for slightly better dispersion compared with solvent casting. The synthesis on PPF nanocomposites has only been reported once, and it involves its reinforcement with 0.1 and 0.3 wt % few layer graphene via in situ polymerization [32]. The incorporation of 0.3 wt % graphene resulted in a 200% increase of the elongation of PPF but did not affect the thermal or the crystallization behavior.

In this work, a series of PPF-based nanocomposites with 1 wt % of nanoclays with different morphologies, i.e., Cloisite^®^-Na and Cloisite^®^-20A, which are nanoplatelets, and HNTs, which are nanotubes, was synthesized for the first time via the in situ transterification and polycondensation method. Although gas permeability was not studied in this work, it is well known that the addition of layered nanoclays in polymers used for film production results in the formation of tortuous paths that gas molecules have to follow, and thus in the further improvement of gas barrier properties [26]. Additionally, an increase in crystallinity is associated with mechanical properties enhancement, better dimensional stability of the final polymeric product, and a decrease in gas permeability [27]. Moreover, the method of in situ preparation of nanocomposites is beneficial compared to melt or solution mixing with respect to the achieved filler dispersion [28]. The present work is aiming to contribute to the field of furanic polyester-based nanocomposites and, more specifically, to clay nanocomposites, which is a topic that has just recently begun to be explored. To the best of our knowledge, there are not any studies reporting PPF-clay nanocomposites and their physicochemical properties so far. Given the above, the objective of this work was the study of the effect of the nanofillers’ forms on the physicochemical properties of PPF, with an emphasis on its crystallization rate, as well as its thermal stability.

## 2. Materials and Methods

### 2.1. Materials

2,5-dimethylfuran-dicarboxylate (DMFD), 1,3-propanediol (PDO), and Tetra-tert-butyl orthotitanate (TBT) catalyst of analytical grade were purchased from Sigma-Aldrich Co., Taufkirchen, Germany. Two types of montmorillonites, Cloisite^®^Na^+^ (MMT) and Cloisite^®^20A (MMT 20A), were purchased by Southern Clay products, Gonzales, TX, USA. Cloisite^®^Na^+^ has interlayer distance 11.7 Å and Cloisite^®^20A, which is modified with dimethyl; dihydrogenatedtallow quaternary ammonium has interlayer distance of 19.2 Å. Halloysite nanotubes (HNT) were purchased from Sigma-Aldrich and possess the chemical formula Al_2_Si_2_O_5_(OH)_4_x2H_2_O and molecular weight 249.19 amu. Their surface area was measured equal to 53.3 m^2^/g with 0.371 mL/g pore volume, and the approximate size of nanotubes was 30–70 nm * 3–8 μm. All other materials and solvents used were of analytical grade and were purchased from Sigma-Aldrich, Taufkirchen, Germany.

### 2.2. Preparation of PPF Nanocomposites

PPF was prepared through two-stage polycondensation in a glass batch reactor [33]. The first step of polycondensation (transesterification) took place by charging DMFD and PDO in a molar ratio of diester/diol = 1/2.2 into the reaction tube of the polyesterification apparatus with 400 ppm of TBT. The mixture was then heated in three steps: firstly, at 160 °C for 1.5 h under argon flow, then at 170 °C for additional 1 h, and finally at 180 °C for 1.5 h. The transesterification step was completed after the whole theoretical amount of methanol was collected, which was removed by distillation from the reaction mixture and collected in a graduated cylinder. In the polycondensation step, vacuum (5.0 Pa) was applied gradually for about 30 min, with the aim of removing the excess of diol for two main reasons: firstly, to avoid excessive foaming of the reaction mixture, and secondly, to minimize the sublimation of the oligomer, which is a potential problem during melt polycondensation. Afterwards, the temperature was increased (1 h) to 220 °C, while stirring speed was also increased to 450 rpm. The reaction continued at this temperature for 1.5 h. Successively, the temperature was increased to 235 °C for 1.5 h and to 250 °C for an additional 1 h.

PPF-based nanocomposites containing 1 wt % of MMT, MMT 20A, and HNT were in situ prepared using the two-stage (esterification and polycondensation) polycondensation method. Each nanofiller was added in PG and subjected to sonication for 15 min aiming to obtain a uniform dispersion. Afterwards, DMFD and TBT, along with the dispersion, were transferred in the reaction apparatus. The reaction continued as described above for the synthesis of neat PPF. After the polycondensation reaction was completed, neat PPF and its nanocomposites were removed from the reactor, milled, and washed with methanol.

### 2.3. Characterization

#### 2.3.1. Intrinsic Viscosity

Intrinsic viscosity [*η*] measurements were performed using an Ubbelohde viscometer (Sigma-Aldrich, Taufkirchen, Germany) at 25 °C in a mixture of phenol/1,1,2,2-tetrachloroethane (60/40, *w*/*w*). Each sample was maintained in the above mixture of solvents at 90 °C for some time to achieve complete dissolution. The solution was then cooled to room temperature and filtered through a disposable Teflon membrane. The intrinsic viscosity of each sample was calculated using the Solomon–Ciuta equation of a single point measurement:

(1) $$\left[\eta \right]={\left[2\left\{t/t_{0}-\ln\left(t/t_{0}\right)-1\right\}\right]}^{0.5}/c$$

in which *c* is the concentration of the solution, *t* is the flow time of the solution, and *t*_0_ is the flow time of pure solvent. For each sample, three different measurements have been done, and the average value was calculated.

The number-average molecular weight (${\bar{M}}_{n}$) of the samples was calculated from intrinsic viscosity [*η*] values, using the Berkowitz equation [34], as was modified in our previous work [35]:

(2) $${\bar{M}}_{n}=3.29\times 10^{4}\;{\left[\eta \right]}^{1.54}$$

#### 2.3.2. Nuclear Magnetic Resonance Spectroscopy (NMR)

^1^H-NMR spectra of polyesters were obtained with an Agilent spectrometer (Agilent Technologies, Santa Clara, CA, USA) operating at a frequency of 400 MHz for protons at room temperature. Deuterated trifluoroacetic acid (DTFA) was used as solvent to prepare solutions of 5% *w*/*v*. The number of scans was 16 and the sweep width was 6 kHz.

#### 2.3.3. Scanning Electron Microscopy (SEM)

SEM was carried out using a JEOL JMS-840A (JEOL, Tokyo, Japan) scanning microscope equipped with an energy-dispersive X-ray (EDX) Oxford ISIS 300 (Oxford Instruments, Abingdon, UK) microanalytical system.

#### 2.3.4. Fourier-Transform Infrared Spectroscopy (FTIR)

FTIR spectra was obtained using a Perkin-Elmer FTIR spectrometer (Perkin Elmer, Waltham, MA, USA), model SPECTRUM 1000, using KBr tablets. The resolution for each spectrum was 2 cm^−1^, and the number of co-added scans was 16. The spectra presented were baseline corrected and converted to absorbance mode.

#### 2.3.5. X-ray Diffraction (XRD)

XRD measurements of the PPF nanocomposites were performed over the 2θ range of 5 to 50°, at steps of 0.05° and scanning speed 1 deg/min, using a MiniFlex II XRD system from Rigaku Co. (Rigaku Company, Tokyo, Japan) with Cu Ka radiation (λ = 0.154 nm).

#### 2.3.6. Differential Scanning Calorimetry (DSC)

The DSC studies were carried out using a TA Instruments temperature modulated DSC (TA Q2000, TA Instruments, New Castle, DE, USA). The instrument was previously calibrated with indium for the accurate determination of heat flow and temperature. The sample mass was kept constant at around 5 mg for all crystallization kinetics tests, while a nitrogen gas flow of 50 mL/min was purged into the DSC cell. The sample and reference pans were of identical mass, with an error ±0.01 mg.

Initially the samples were cooled to 0 °C and then heated at a rate of 20 °C/min to a temperature 40 °C higher than the melting temperature. The samples were held at this temperature for 5 min in order to erase any thermal history and were then cooled back to the isothermal crystallization temperature (*T*_c_) with the highest achievable rate. At that temperature, the sample was held for a time *t*_c_, which was selected based on the recommendations from Muller and co-workers, that crystallization time should be 5 times the time to reach the minimum of the exothermic signal generated as a function of time at *T*_c_ [36]. After the samples were isothermally crystallized at different temperatures, they were re-heated at a rate of 20 °C/min up to 230 °C, in order to study their melting behaviour after isothermal crystallization.

#### 2.3.7. Thermogravimetric Analysis (TGA)

TGA measurements were carried out with a SETARAM SETSYS TG-DTA 16/18 instrument (Setaram instrumentation, Lyon, France). Samples (4.3 ± 0.2 mg) were placed in alumina crucibles, with the temperature program set from ambient temperature up to 600 °C in a 50 mL/min nitrogen flow, under the rate of 10 °C/min. A baseline experiment was performed prior to each sample measurement, and it was subsequently subtracted by the second one, in order to eliminate the buoyancy effect. Regarding the kinetic analysis study, measurements were carried out at four different heating rates, namely, 5, 10, 15, and 20 °C/min. The repeatability of the experimental procedure was ensured by the accomplishment of multiple measurement runs of each sample.

#### 2.3.8. Pyrolysis-Gas Chromatography/Mass Spectroscopy

For Py-GC/MS analysis of polyesters, a very small amount of each material is “dropped” initially into the “Double-Shot” EGA/PY-3030D Pyrolyzer (Frontier Laboratories Ltd., Fukushima, Japan) using a CGS-1050Ex (Frontier Laboratories Ltd., Fukushima, Japan) carrier gas selector. For Evolved Gas Analysis (EGA), the furnace temperature was programmed from 50 to 700 °C with a heating rate of 20 °C/min, using He as purge gas and air as cooling gas. For pyrolysis analysis (flash pyrolysis), each sample was placed into the sample cup, which afterwards fell free into the Pyrolyzer furnace. The pre-selected pyrolysis temperatures were 360 and 400 °C, and the GC oven temperature was heated from 70 to 300 °C at 10 °C/min. This temperature was selected based on the EGA. Sample vapors generated in the furnace were split (at a ratio of 1/50): a portion was moved to the column at a flow rate of 1 mL/min, pressure 53.6 kPa, and the remaining portion exited the system via the vent. The pyrolyzates were separated using temperature programmed capillary column of a Shimadzu QP-2010 Ultra Plus (Shimadzu, Kyoto, Japan) gas chromatograph and analyzed by the mass spectrometer MS-QP2010SE of Shimadzu (Shimadzu, Kyoto, Japan) use 70 eV. Ultra ALLOY^®^ metal capillary column from Frontier Laboratories LTD (Fukushima, Japan) was used containing 5% diphenyl and 95% dimethylpolysiloxane stationary phase, column length 30 m, and column ID 0.25 mm. For the mass spectrometer, the following conditions were used: Ion source heater 200 °C, interface temperature 320 °C, vacuum 10^−4^–10^0^ Pa, *m*/*z* range 45–500 amu, and scan speed 10,000. The chromatograph and spectra retrieved by each experiment are subject to further interpretation through Shimadzu and Frontier post-run software.

## 3. Results and Discussion

### 3.1. Preparation of PPF Nanocomposites

**Scheme 1:**
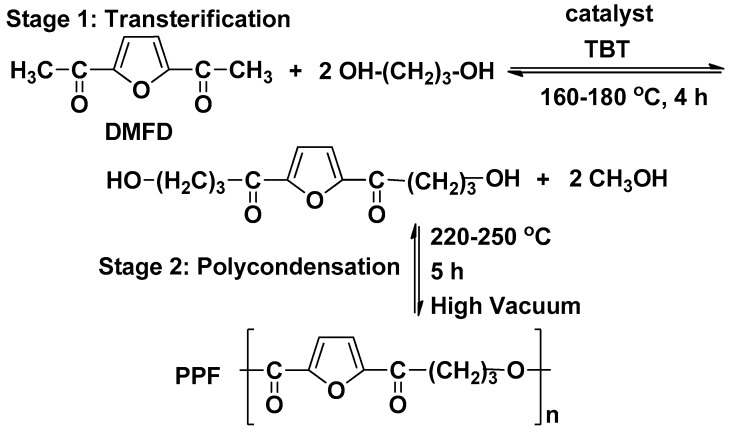
Synthesis of PPF.

The PPF nanocomposites were synthesized via the two-step melt polycondensation method. The reaction procedure is presented in Scheme 1. The colour of the final materials depended on their composition. Neat PPF was light brown, while PPF/MMT and PPF/MMT 20A were brown, with PPF/MMT being the darker of the two. As for PPF/HNT, the material was yellow. The physical state of the materials was solid.

**Figure 1:**
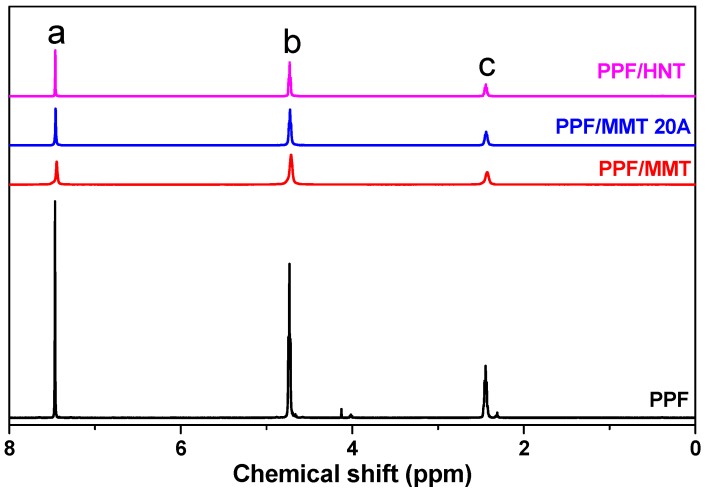
^1^H-NMR spectra of PPF and its nanocomposites with MMT, MMT 20A, and HNT.

**Scheme 2:**
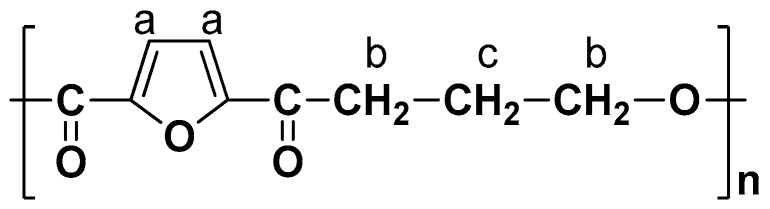
PPF structure and ^1^H-NMR peak assignments.

The intrinsic viscosity values of PPF and its nanocomposites with MMT, MMT 20A, and HNT were 0.50, 0.47, 0.45, and 0.41, respectively. The structure of the prepared materials was verified by ^1^H-NMR spectroscopy, presented in Figure 1, while the peak assignments are presented in Scheme 2. In the spectrum of PPF neat, the furan ring protons (a) are the most deprotected, due to the π electron system of the ring and the carbonyl groups, and they appear at 7.45 ppm. The propylene glycol protons appear at lower values, at 4.75 ppm for the protons near the oxygen atoms (b), which are the most deprotected, and at 2.44 ppm for the (c) protons. The above results are in accordance with our previous work [19]. The same peaks were recorded for the nanocomposites, thus confirming that the addition of the clays had no effect on the molecular structure of the prepared polyesters.

### 3.2. Morphological and Structural Characterization

**Figure 2:**
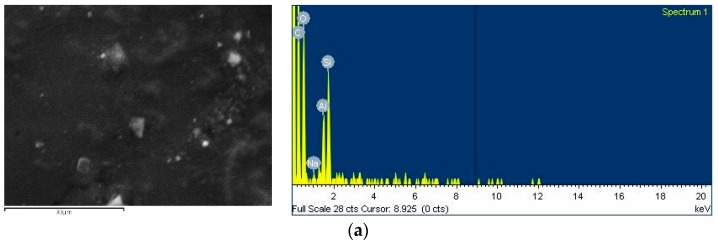

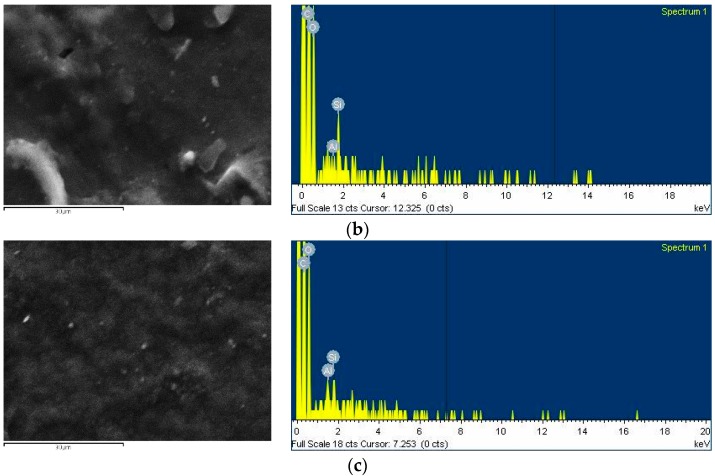
SEM micrographs and EDX spectra of (**a**) PPF/MMT, (**b**) PPF/MMT 20A, and (**c**) PPF/HNT nanocomposites. The scale bar in the micrographs is 30 μm.

SEM micrographs and EDX analysis spectra were obtained for the PPF/clay nanocomposites to confirm the presence of the nanofillers in the polymeric matrix. The results are presented in Figure 2. All clay fillers appear as small white dots on the images and were identified by the elemental analysis that confirmed the existence of silicon and aluminum. In the case of PPF/MMT, sodium was also detected, which agrees with the provider’s description of Cloisite^®^Na^+^. Even if SEM micrographs cannot reveal the extent of exfoliation or intercalation of the clays, they provide useful information about their dispersion and possible aggregation. Comparing the dispersion of the 3 nanofillers, MMT appears to be slightly agglomerated, since larger white spots appear on its surface. In contrast, MMT 20A and HNT are more finely dispersed, with less obvious aggregates. This better dispersion can be attributed to the larger interlayer distance of MMT 20A that can allow the easier penetration of the macromolecular chains in between the clay’s layers during polymerization, and to the more hydrophobic nature of HNTs that make them more compatible with the PPF matrix, as well as their morphology that constitutes individual tubes rather than stacked platelets that tend to aggregate.

**Figure 3:**
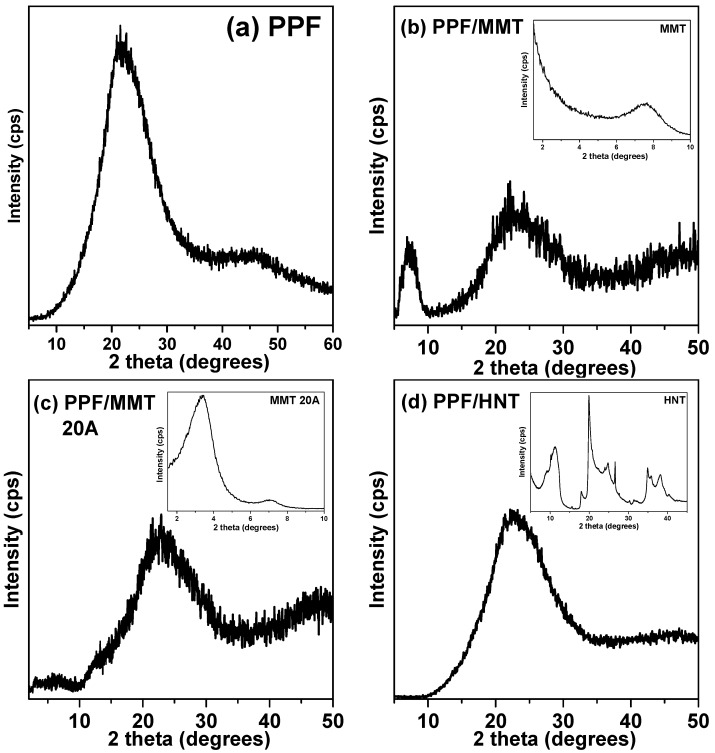
WAXD patterns of the nanofillers and their nanocomposites with PPF.

X-ray diffraction was used to evaluate the dispersion of the nanofillers in the PPF matrix, along with SEM. Layered silicates have a diffraction peak that corresponds to the d-spacing between the layers, and when incorporated in polymeric matrices, the emerging or not of this peak is related to its dispersion, which can be intercalated or exfoliated. MMT Na shows the diffraction peak of the (001) crystal plane at 2*θ* = 7.6° that corresponds to d-spacing of about 11.6 Å; MTT 20A has a peak of the same crystal plane at 2θ = 3.4° with a larger d-spacing of about 26 Å due to its modification, and, finally, HNT’s (001) plane appears at 2θ = 11.24° with *d* = 7.9 Å (Figure 3).

The diffraction pattern of PPF/MMT has one peak at 2θ ≈ 7.2° due to the presence of MMT that suggests an intercalated structure. The d-spacing of MMT increased from 11.6 Å to 12.3 Å because of the insertion of polymeric chains between the layers. The nanocomposite PPF/MMT 20A presents a very small diffraction peak at 2*θ* ≈ 2.1°, which corresponds to an increase of the d-spacing of MMT 20A from 26 Å to 42 Å; thus, the extent of penetration of the PPF chains in the interlayer space and the subsequent separation of the layers is more pronounced, possibly resulting in both intercalated and partially exfoliated structure of the particular nanocomposite [37,38]. Finally, the nanocomposite PPF/HNT does not present any diffraction peaks associated with the nanofillers, suggesting its fine dispersion in the polymeric matrix. However, it is expected that some aggregates might also present that cannot be detected through WAXD due to the low filler content. These observations are in agreement with the conclusions drawn by the SEM study. MMT and modified MMT platelets were found to be predominately intercalated in concentrations 2 wt % and 4 wt % in PEF nanocomposites prepared by solution mixing and melt compounding [30,31].

**Figure 4:**
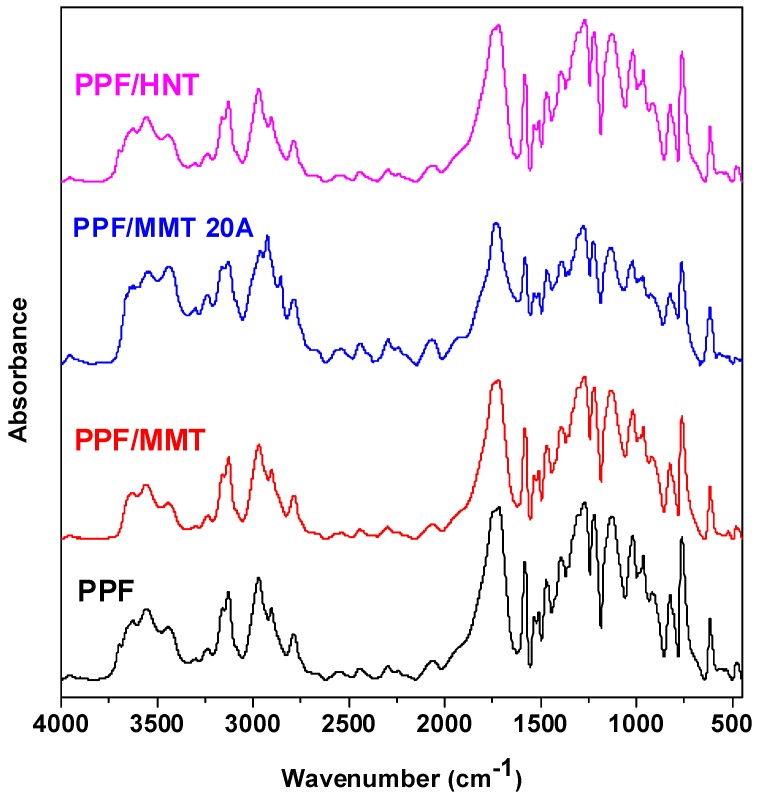
FTIR spectra of PPF and its nanocomposites.

The FTIR spectra of the materials is shown in Figure 4, and they can confirm the successful synthesis of the polyesters. More specifically, characteristic peaks of the furan ring at 3160 and 3130 cm^−1^ that correspond to C_sp2_-H bond and at 1580 and 1530 cm^−1^ that correspond to the double bond of the furan ring are clearly visible. Also, characteristic peaks at 2962, 2899, and 2786 cm^−1^ corresponding to C_sp3_-H bond of propylene glycol and at 1730 cm^−1^ corresponding to the ester bond verify the structure of the materials in question and are in accordance with similar studies [39]. As in the ^1^H-NMR studies, there are not any clear peaks associated with the nanofillers, presumably due to the high molecular weight of the samples and the low filler content of the nanocomposites (1 wt %). However, for PPF/MMT 20A, an increase in the intensity of the peak at 3435 cm^−1^ is observed, in addition to a new peak at 2919 cm^−1^. These two peaks are probably assigned to the quaternary ammonium molecules, which are used for the modification of the montmorillonite, thus differentiating this spectrum from the rest.

### 3.3. Crystallization Study

#### 3.3.1. Isothermal Crystallization

**Figure 5:**
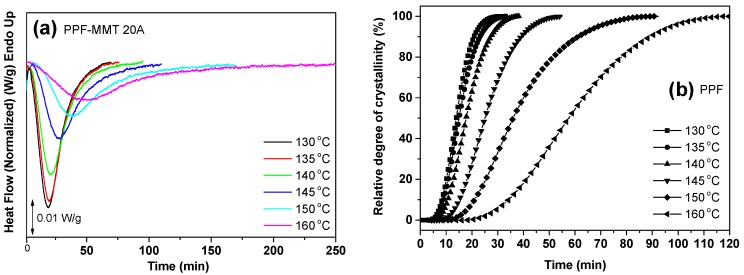
(**a**) Isothermal crystallization peaks recorded at different crystallization temperatures, for the PPF-MMT sample (**b**) evolution of the relative degree of crystallinity versus time for the PPF sample.

**Figure 6:**
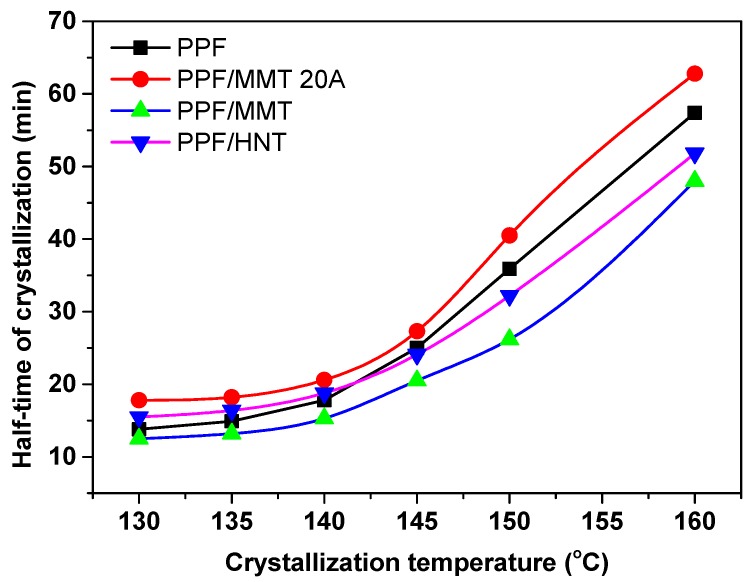
Dependence of the half-time of crystallization to isothermal crystallization temperature, for neat PPF and the nanocomposites.

The crystallization phenomenon is known to be accompanied by significant heat release. In this work, we assumed that the evolution of crystallinity can be proportional to the evolution of heat that is released while crystallization is taking place, and, therefore, we obtained the relative degree of crystallinity from the following equation [29]:

(3) $$X(t)=\frac{\int_{0}^{t}(dH_{c}/dt)dt\;}{\int_{0}^{\infty }(dH_{c}/dt)dt}$$

in which *dHc* stands for the enthalpy of crystallization during an infinitesimal time interval *dt*. The limits *t* and ∞ on the integrals are used to denote the elapsed time during the course of crystallization and at the end of the crystallization process, respectively. The isothermal crystallization experiments for PPF and the clay-reinforced nanocomposites were performed at a range of temperatures, from 130 to 160 °C. As it can be seen from Figure 5a, as the supercooling (i.e., the difference between the melting and crystallization temperature) decreases, the crystallization phenomenon proceeds at slower rates, and the exothermal peaks of crystallization become broader. The results from the evolution of the relative degree of crystallinity obtained from Equation (3), only for the PPF sample (for brevity reasons), can be seen in Figure 5b. The half time of crystallization is an indication of the crystallization rate, and it can be obtained from the S-shaped curves of Figure 5b. According to the results presented in Figure 6, the *t*_1/2_ increases almost exponentially with increasing crystallization temperature. It is interesting to notice that the sample filled with MMT-20A does not display any enhanced crystallization rates, but, on the contrary, the presence of MMT-20A slightly delays the crystallization phenomenon. On the other hand, the sample filled with MMT presents faster crystallization rates than the neat polymer, most possibly due to the absence of quaternary ammonium that is present between the layers of MMT 20A. Finally, the halloysite nanotubes are only active at higher isothermal crystallization temperatures (*T*_c_ > 145 °C), while at low *T*_c_, they are almost inert.

#### 3.3.2. Application of the Avrami Theory

**Figure 7:**
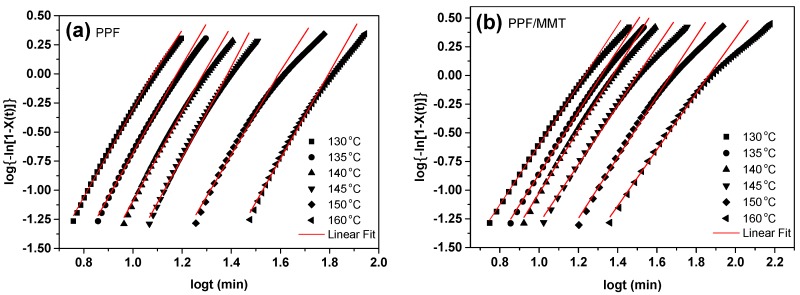
Avrami plots for the isothermal crystallization of PPF and PPF-MMT samples.

The well-known Avrami equation was employed to analyse further the results from the isothermal crystallization. According to the Avrami theory, the time-dependent crystallinity *X*(*t*) obtained from an isothermal crystallization process can be expressed as [40,41]:

(4) $$X\left(t\right)=1-\exp\left(-Kt^{n}\right)$$

in which *n* is the Avrami exponent that is a function of the nucleation process and *K* is the growth function, which is dependent on the nucleation and crystal growth. Both Avrami parameters can be calculated by fitting the experimental data to the double logarithmic form of Equation (4). In Figure 7a,b, plots of log[−ln(1−*X*(*t*))] versus log(*t*) can be seen for the PPF and PPF-MMT 20A samples, respectively. As it can be seen, straight lines are obtained from the fitting for each isothermal crystallization temperature (at the 5–90% range). From the intercept and the slope of each line, log*K* and n can be calculated. The fitting values for the Avrami parameters can be seen in Table 1. The values of the Avrami exponent did not display any clear trend, however; *n* was smaller in the case of the nanocomposite samples (ranging between 2–3), compared to neat PPF, in which n was in the range of 3.5–3.8. This is an indication that the nucleation mechanism was altered to heterogeneous in the case of the nanocomposites. An *n* value around 3 indicates three-dimensional growth. Moreover, as it was expected, the values of *K* decrease with increasing crystallization temperature, while the differences between the samples agree with the results obtained from the half-time of crystallization.

#### 3.3.3. Melting Behaviour

**Figure 8:**
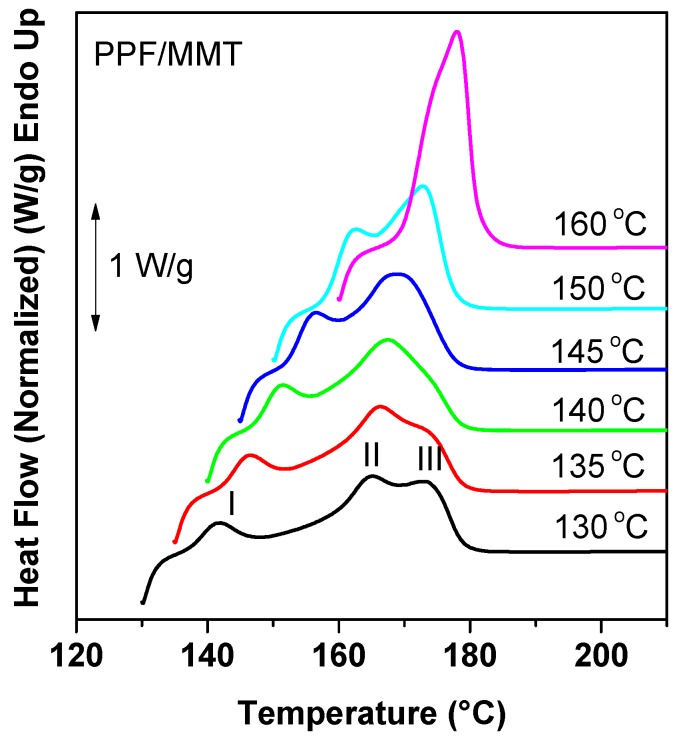
DSC traces for PPF-MMT-Na sample, crystallized at different temperatures. The applied heating rate was 20 °C/min.

In accordance with the majority of furan-based polyester, the DSC traces after isothermal crystallization revealed the multiple melting behaviour of the neat polymer and the nanocomposite samples. In Figure 8, the effect of the isothermal crystallization on the melting behaviour can be seen. For the samples crystallized from the melt at temperatures equal or lower than 140 °C, three melting peaks can be clearly observed. The low temperature peak (I) appeared a few degrees above the isothermal crystallization temperature and can be related to the melting of secondary crystals that are formed during the isothermal crystallization. This peak was observed for all samples except for the one crystallized at 160 °C, in which slow crystallization leads to the formation of crystals with high thermal stability and crystalline perfection. Regarding the peaks II and III, it can be seen that with the increase of *T*_c_, peak III decreases in intensity and eventually merges with peak II. This is an indication that peak III is related to the melting of crystals that are formed after partial melting and recrystallization during the heating scan. Moreover, peak II is related to the melting of primary crystals that are generated during the isothermal crystallization phenomenon. These findings prove that the materials follow the well-known melting-recrystallization-re-melting scheme that is well accepted for numerous polyesters [42,43]. 

### 3.4. Thermal Degradation

**Figure 9:**
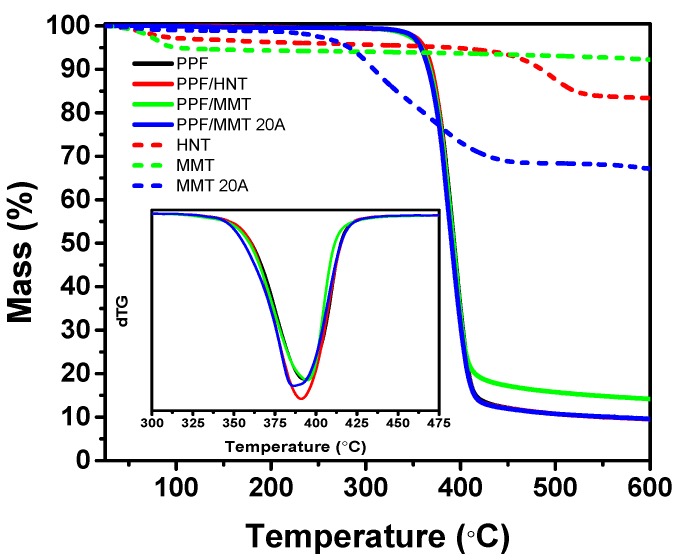
TG and dTG curves (shown inset) of PPF and its nanoclay-based nanocomposites recorded at a 10 °C/min heating rate in nitrogen atmosphere.

Thermal stability of PPF and its nanocomposites with clays is of great importance, since it directly affects their processing temperatures and thereby their applications [8]. Thermal degradation of PPF and its clay-based nanocomposites was studied by means of thermogravimetric analysis. Figure 9 displays remaining Mass (%) and Derivative Mass loss (dTG) (shown inset) curves of all the PPF-based samples, from which it can be deduced that degradation is carried out as a one-step process for all the studied samples and that no remarkable mass loss has occurred until 300 °C, proving the strong thermal stability of PPF-based specimens. In the same Figure, thermogravimetric curves of the nanoclays are also exhibited (dashed curves) for comparative purposes.

Mass curves of all samples are seemingly almost identical up to ~400 °C, consisting of a one-step procedure and obtaining the same curve shape. However, a closer and more careful look, especially at the dTG curve, reveals divergences between them. More specifically, though the decomposition of all samples is carried out in the same manner up to 340 °C, the decomposition of PPF/MMT 20A, starting from 340 °C, takes place in a sharper manner compared to the rest samples, resulting in a slightly accelerated degradation procedure. This is not unexpected, since analogous behavior has been observed to samples containing modified nanoclays. Chrissafis et al. [2] have studied thermal degradation of PCL nanocomposites with MMT 20A under dynamic conditions, and they have reported that modified montmorillonite has accelerated the polymer’ decomposition, due to different reactions that can be induced by the reactive groups on the nanoparticles’ surface. More specifically, the effect of accelerated decomposition may be attributed to the decomposition of the ammonium salt that was used in order for the montmorillonite to be modified or to the aminolysis reaction that ammonium salt may have induced on the macromolecules of PPF.

Furthermore, the behavior of both PPF/HNT and PPF/MMT is very close to the correspondence of PPF neat up to ~400 °C, in which the corresponding nanoclays act nearly as inert fillers, since marginal variations in terms of thermal stability are observed. The process of dehydroxylation, as well as moisture entrapped in those two nanoclays—as evidenced by the dotted TGA curves in Figure 9—may prevent them from decelerating the thermal degradation of PPF [3,4].

The key parameters for the evaluation of thermogravimetric measurements, such as the temperature at 5% and 10% weight loss (*T*_5%_ and *T*_10%_, respectively) and the temperature that corresponds to the maximum decomposition rate (*T*_d,max_) are all summarized in Table 2.

The temperature that corresponds to the maximum decomposition rate for poly(propylene furanoate) is *T*_d,max_ = 393 °C, which is close to reported studies, such as 387 ± 1 °C according to Genovese at al. [44] and 396 °C according to Papageorgiou et al. [11]. Furthermore, *T*_d,max_ of PPF is slightly lower than that of PPF/MMT (*T*_d,max_ = 395 °C) and slightly higher than those of PPF/HNT (*T*_d,max_ = 391 °C) and PPF/MMT 20A (*T*_d,max_ = 386 °C). Moreover, the temperature that corresponds to 5% mass loss of PPF is slightly lower than that of PPF/HNT and slightly higher than those of both the PPF/MMT nanocomposites. The same trend applies to the 10% mass loss of all the studied samples. Nonetheless, these differences are quite small.

### 3.5. Thermal Degradation Mechanism Study by Py/GC-MS

**Figure 10:**
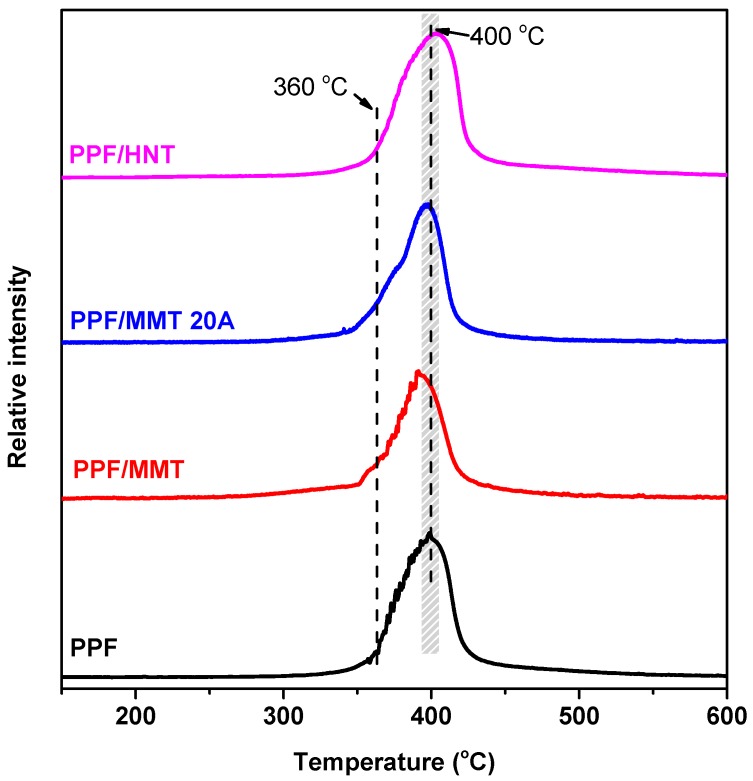
EGA pyrograms of PPF and its nanocomposites.

**Figure 11:**
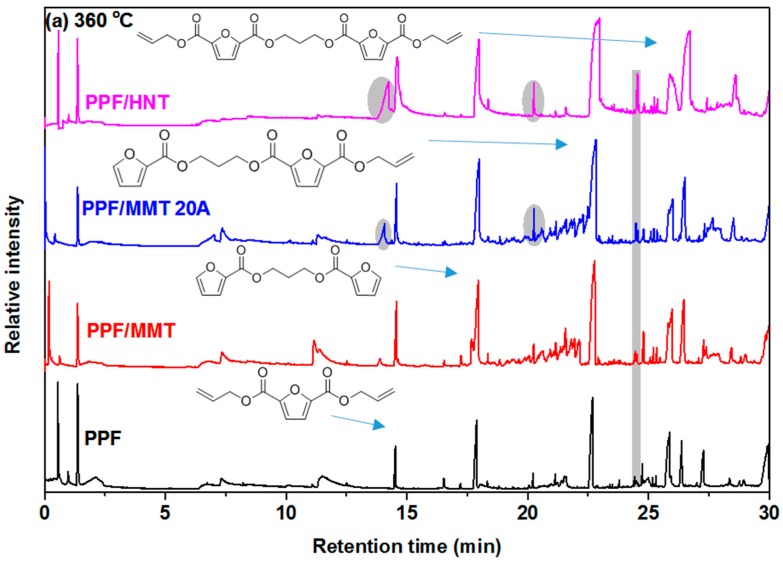

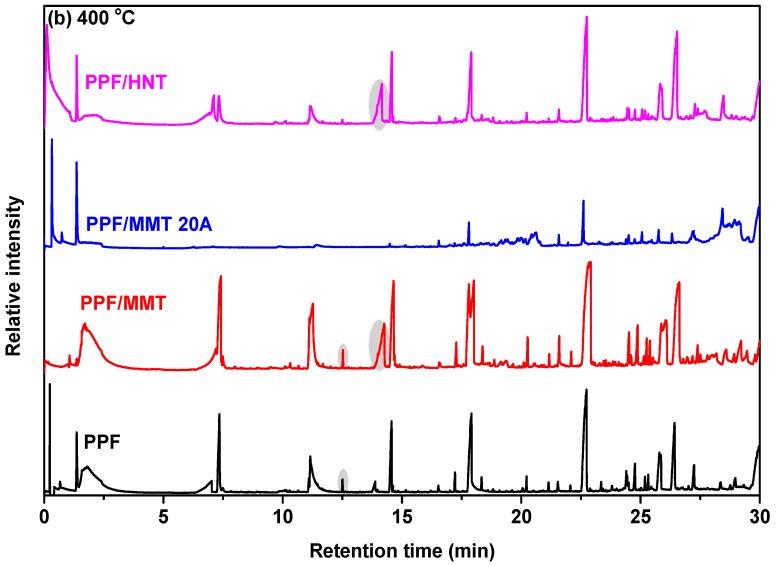
Gas chromatographs of PPF and its nanocomposites after pyrolysis at (**a**) 360 °C and (**b**) 400 °C.

The evaluation of the effect of the nanoclays on the thermal degradation mechanism of PPF was conducted by Py/GC-MS. First, the polyesters were subjected in EGA analysis, during which they were pyrolyzed with an increasing temperature program; the evolved gases were inserted in the GC apparatus, travelled through an inert column, and were finally detected by the mass spectrometer. The resulting pyrograms are presented in Figure 10. The temperature range in which pyrolysis products are released is about 350–450 °C, with no significant differences between the samples, which is in agreement with the TGA measurements of Figure 9. The flash pyrolysis (single-shot) temperatures of 360 °C and 400 °C were selected by the EGA pyrogram and represent the beginning and the maximum rate of degradation, respectively. During flash pyrolysis, the samples were pyrolyzed in the two preselected temperatures, separated by a capillary column in the GC apparatus, and finally identified with their MS spectra. The gas chromatographs of PPF and its nanocomposites with clays after pyrolysis at 360 °C and 400 °C are presented in Figure 11.

The chromatographs after pyrolysis at 360 °C exhibit fewer peaks, therefore the degradation mechanism is simpler in these temperatures compared with 400 °C. The major pyrolysis products of neat PPF at 360 °C in Rt = 1.36, 14.52, 17.89, 22.69, 25.88, 26.38, and 27.28 min were identified as CO_2_, diallyl furan-2,5-dicarboxylate, propane-1,3-diyl bis(furan-2-carboxylate), 2-allyl 5-(3-((furan-2-carbonyl)oxy)propyl) furan-2,5-dicarboxylate, 5-((3-((5-(propoxycarbonyl)furan-2-carbonyl)oxy)propoxy)carbonyl)furan-2-carboxylic acid, and 5-diallyl O’^2^,O^2^-propane-1,3-diyl bis(furan-2,5-dicarboxylate), which are mostly compounds with vinyl end groups. In the same temperature, the nanocomposites with MMT, MMT 20A, and HNT exhibit an additional peak in Rt = 13.90, 14.08, and 14.25 min, respectively, which were identified as 5-((allyloxy)carbonyl)furan-2-carboxylic acid, an important increase in the intensity of the peak at Rt ≈ 20.25 min, which was identified as 2-(3-((furan-2-carbonyl)oxy)propyl) 5-methyl furan-2,5-dicarboxylate, a methyl ester terminated compound, and an increase in the intensity of the peak at Rt ≈ 24.50 min, which could possibly be a molecule that results from random radical disproportionation, since it does not match any known furanic polyester degradation products; however it cannot be confirmed. The main differences between the pyrolysis products of PPF with its nanocomposites at 400 °C is the emerging of the peak in Rt = 14.28 min that was identified as 5-((allyloxy)carbonyl) furan-2-carboxylic acid. It is also noteworthy that the nanocomposites PPF/MMT 20A and PPF/HNT present a simpler chromatograph that the other samples at 400 °C, which could indicate a deceleration effect on thermal degradation. All the identified products are presented in Table S1.

**Scheme 3:**
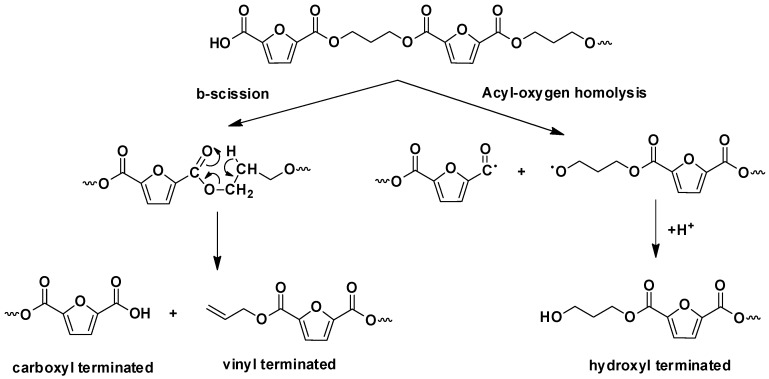
Main degradation mechanisms of PPF.

For simplification reasons, most pyrolysis products can be assorted into vinyl-carboxyl terminated compounds and hydroxyl-terminated compounds. These two types are derived from two different degradation mechanism pathways that were studied in depth in previous publications of our group [45,46,47,48,49,50,51], heterolytic (β-hydrogen scission) and homolytic (acyl-oxygen and alkyl-oxygen scission) routes. β-scission is the dominant degradation mechanism that results in vinyl and carboxyl terminated compounds, while homolysis can result in hydroxyl-, aldehyde-, and alkyl-terminated compounds. The latter can also be generated from hydrogenation of vinyl terminated degradation products as well. Both pathways are presented in Scheme 3. 

The form of the chromatographs is also pyrolysis temperature-dependent. After pyrolysis at 400 °C, the peaks at Rt = 5.00–15.00 min become more pronounced. These peaks at ~7.30, ~11.00, ~12.50, and ~13.90 min were identified as allyl furan-2-carboxylate, di(furan-2-yl) methanone, 1,2-di(furan-2-yl)ethane-1,2-dione, and 5-((allyloxy)carbonyl)furan-2-carboxylic acid, respectively. The two vinyl terminated compounds are more pronounced due to the more extensive heterolytic scission along the macromolecular chain that is a result of the higher pyrolysis temperature, while the other two ketones could be produced from the coupling of radicals created during the homolytic degradation mechanism presented in Scheme 3. This observation indicates that the homolytic pathways are secondary mechanisms that are favored in higher pyrolysis temperatures and therefore need more thermal energy to occur.

## 4. Conclusions

PPF nanocomposites containing 1% MMT, MMT-20A, and HNTs were in situ prepared via melt polycondensation. Molecular characterization of the samples showed that a good control of the nanocomposite preparation method was achieved. XRD patterns indicated the exfoliation and a fine dispersion of the nanocomposites into the polymer matrix. DSC results showed that while MMT and HNTs act as a nucleating agent, modified MMT-20A has the opposite effect, most possibly due to the presence of quaternary ammonium between its layers. TGA results showed that the samples present good thermal stability, with no significant mass loss up to 300 °C but with slightly accelerated degradation rates compared with neat PPF above that temperature. Finally, Py/GC-MS results showed that there are two degradation mechanism pathways, the heterolytic (β-scission) and the homolytic (acyl-oxygen and alkyl-oxygen scission), and that β-scission is the dominant degradation mechanism, while the homolytic route is a secondary pathway that requires more thermal energy to occur. Although further studies are needed to fully investigate the potential of PPF nanocomposites with aluminosilicate clays, such as their gas barrier properties, the present work can serve as a starting point for additional research, as the scientific and industrial communities’ interests in PPF are increasing. 

## Figures and Tables

**Table 1:** Avrami parameters; *n* and *K*, obtained from fitting the experimental data from isothermal crystallization for PPF and nanocomposites, with the Avrami equation.

**Temperature**	**PPF**			**PPF/MMT 20A**	
	***n***	***K*** **(min^−*n*^)**		***n***	***K*** **(min^−*n*^)**
130	3.7	1 × 10^−4^	130	2.6	6.3 × 10^−4^
135	3.6	4 × 10^−5^	135	2.7	3.1 × 10^−4^
140	3.8	1.2 × 10^−5^	140	2.7	2 × 10^−4^
145	3.7	5 × 10^−6^	145	2.5	1.2 × 10^−4^
150	3.5	2 × 10^−6^	150	2.6	4 × 10^−5^
160	3.7	2 × 10^−7^	160	2.4	2. × 10^−5^
**Temperature**	**PPF/MMT**			**PPF/HNT**	
	***n***	***K*** **(min^−*n*^)**		***n***	***K*** **(min^−*n*^)**
130	2.5	7 × 10^−3^	130	2.8	3 × 10^−4^
135	2.7	1 × 10^−4^	135	2.6	5 × 10^−5^
140	2.6	5.5 × 10^−5^	140	2.5	1.4 × 10^−5^
145	2.4	9 × 10^−6^	145	2.3	3.1 × 10^−6^
150	2.6	5.2 × 10^−6^	150	2.6	1.1 × 10^−6^
160	2.7	6.1 × 10^−7^	160	2.7	1 × 10^−7^

**Table 2:** TGA results of all the studied samples.

Sample	*T*_d,5%_ (°C)	*T*_d,10%_ (°C)	*T*_d,max_ (°C)
PPF	361	369	393
PPF/HNT	362	370	391
PPF/MMT	359	368	395
PPF/MMT 20A	358	366	386

## Supplementary Materials

The following are available online at http://www.mdpi.com/2073-4360/10/9/937/s1, Table S1. Identified possible pyrolysis products of PPF and its nanocomposites.
