# Supplementary material for: Synthesis and Characterization of In-Situ-Prepared Nanocomposites Based on Poly(Propylene 2,5-Furan Dicarboxylate) and Aluminosilicate Clays
Source: Polymers (Basel). 2018 Aug 23;10(9):937. doi: 10.3390/polym10090937 (PMC6403680; doi:10.3390/polym10090937)
Supplement: Supplementary file 1 [file polymers-10-00937-s001.pdf]

polymers

MDPI

Article

Synthesis and Characterization of In-Situ-Prepared Nanocomposites Based on Poly(propylene 2,5-furan dicarboxylate) and Aluminosilicate Clays

Lazaros Papadopoulos [1], Zoi Terzopoulou[1], Dimitrios Bikiaris[1], Dimitra Patsiaoura[2], Kostantinos Chrissafis[2], Dimitrios G. Papageorgiou[3] and George Papageorgiou [4*]

[1] Laboratory of Polymer Chemistry and Technology, Department of Chemistry, Aristotle University of Thessaloniki, GR-541 24 Thessaloniki, Greece; lazaros.geo.papadopoulos@gmail.com (L.P.), terzozoi@chem.auth.gr (Z.T.), dbic@chem.auth.gr (D.B.)

[2] Solid State Physics Section, Physics Department, Aristotle University of Thessaloniki, GR-541 24 Thessaloniki, Greece; dpatsi@physics.auth.gr (D.Pat.); hrisafis@physics.auth.gr (K.C.)

[3] School of Materials and National Graphene Institute, University of Manchester, Oxford Road, M13 9PL Manchester, United Kingdom; dimitrios.papageorgiou@manchester.ac.uk

[4] Department of Chemistry, University of Ioannina, P.O. Box 1186, GR-45110 Ioannina, Greece; gzpap@cc.uoi.gr (D.G.P.)

* Correspondence: gzpap@cc.uoi.gr; Tel.: +30-2651008354

Received: date; Accepted: date; Published: date

Supplementary Materials

polymers MDPI

Table S 1. Identified possible pyrolysis products of PPF and its nanocomposites.

PPF		PPF/MMT		PPF/MMT 20A		PPF/HNT			Possible product
Rt (min)		Rt (min)		Rt (min)		Rt (min)			
360 ° C	400 ° C	360 ° C	400 ° C	360 ° C	400 ° C	360 ° C	400 ° C	Mw (amu)	
-	0.24	0.17	-	-	0.32	-	0.11	44	CO_2 or acetaldehyde
0.55	-	-	-	-	-	0.56	-	76	1,3-propanediol
1.36	1.36	1.36	-	1.36	1.36	1.36	1.36	44	CO_2 or acetaldehyde
-	1.8	-	1.66	-	-	-	-	68	
6.74	7.02	6.82	7.20	7.03	7.01	-	7.12	112	
7.33	7.35	7.36	7.41	7.34	-	-	7.33	152	
11.09	11.09	11.15	11.18	11.10	-	-	-	162	
-	11.16	-	11.27	-	-	-	11.16	170	or
-	12.51	-	12.53	-	-	-	12.50	190	
-	13.88	13.90	14.26	14.08	-	14.24	14.16	196	

14.52	14.57	14.57	14.65	14.58	14.49	14.61	14.58	236	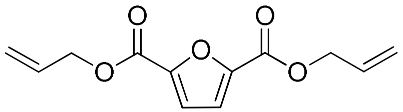
-	-	17.67	17.81	-	-	-	-	254	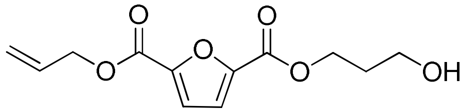
17.89	17.91	17.95	18.01	18.00	17.81	17.98	17.97	264	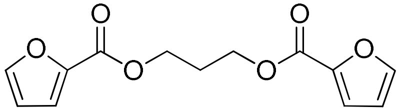
20.22	-	20.24	-	20.27	-	20.27	-	322	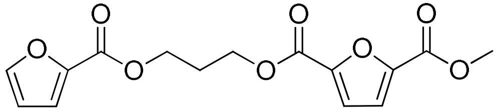
21.52	21.54	21.56	21.59	21.56	21.58	21.57	21.58	336	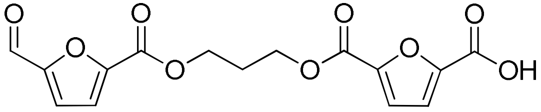
22.69	22.73	22.77	22.91	22.82	22.61	22.96	22.75	348	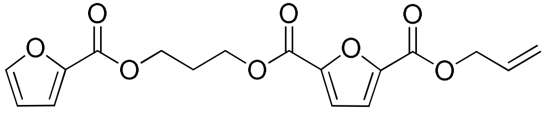
24.42	24.50	24.45	24.52	24.49	24.51	24.55	24.52	388	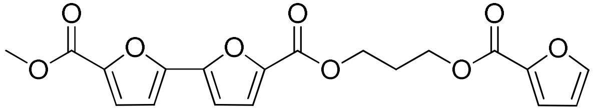
25.18	25.19	25.21	25.26	25.23	-	25.25	25.20	366	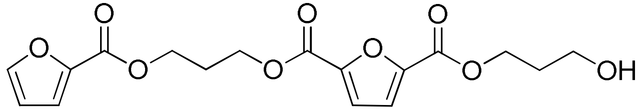
25.31	25.32	25.35	25.40	25.36	-	25.38	25.33	350	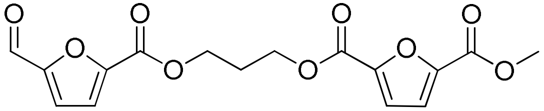
25.88	25.85	25.99	26.05	26.01	25.76	25.90	25.87	392	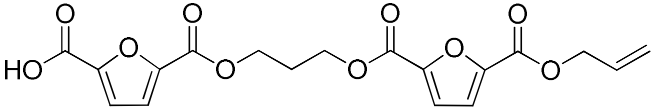

26.38	26.43	26.48	26.63	26.52	26.32	26.71	26.54	432	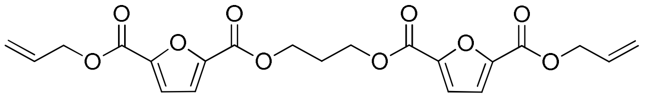
27.28	27.25	27.29	27.39	27.33	-	27.43	27.28	436	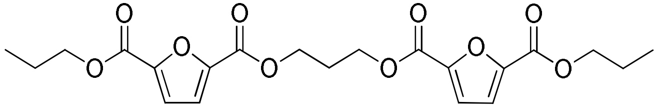
28.37	-	28.45	-	28.53	28.44	28.61	28.48	488	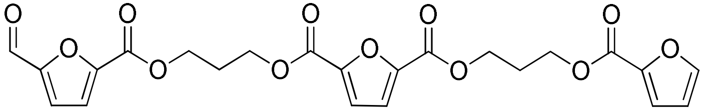
29.97	29.98	-	-	-	-	-	-	460	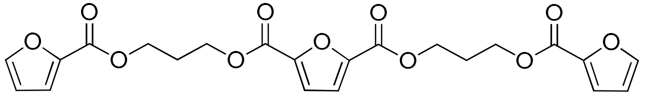
